# Virginia State Veterinary Medical Association

**Published:** 1896-08

**Authors:** George C. Faville, Thomas M. Sweeny, W. H. Harbaugh

**Affiliations:** Secretary; Richmond, Secretary-elect.; President


### VIRGINIA STATE VETERINARY MEDICAL ASSOCIATION.

THE third annual meeting was held at Norfolk, June 24, 1896, President Dr. W. H. Harbaugh in the chair, and Secretary Dr. George C. Faville at his post. The following members were present: Drs. Harbaugh, Faville, Gilchrist, Niles, Drake, Dixon, McCulloch, Bannister, Curtice, and Sweeny. Health-officers and laymen of Newport News and the city were also present. Drs. F. S. Roop, Blacksburg; E. E. Bowen, Lynchburg; John Rome, Colonial Beach; and A. J. Burkholder, Staunton, were elected to membership.

After listening to the retiring president's address and transacting routine business, the following officers were unanimously elected for the ensuing year: Dr. George C. Faville, Norfolk, President; Dr. W. T. Gilchrist, Norfolk, First Vice-President; Dr. Charles McCulloch, Howardsville, Second Vice-President; Dr. Thomas M. Sweeny, Richmond, Secretary; and Dr. A. J. Burkholder, Staunton, Treasurer.

The Association then took a recess and enjoyed the hospitality of Dr. George C. Faville at his elegant home at Atlantic City. Later they were driven to Lambert's Point piers to witness the inspection, tagging, etc., of export cattle, previous to their embarkation for England, after which the members returned to Norfolk.

The Association reconvened at 4.45 P.M. During the afternoon session the following papers were read: Dr. H. S. Drake, "Navicular Disease;" Dr. Cooper Curtice, "Eradication of the Cattle Tick." Dr. W. H. Harbaugh also read an interesting paper, the title of which was "Report of a Subnormal Temperature." All the papers were fully discussed.

The veterinarians of the State are actively engaged in a brisk fight against bovine tuberculosis. The disease was fully discussed, after which the following resolutions were passed: "We, the Virginia State Veterinary Medical Association, fully indorse the work being done in this State in regard to the prevention of products of tuberculous animals being sold as human food; and we desire to inform the public that the tuberculin-test is the only reliable means of diagnosing tuberculosis in cattle; and we further assure the public that when tuberculin is properly used it has positively no deleterious effect on healthy cows or their milk. We further declare that milk or meat from tuberculous animals is impure and unfit for human food, no matter to what extent the animal may be affected. It is the opinion of this Association that the National Government should adopt radical methods to exterminate this disease and prevent any more of it being introduced from abroad by the importation of thoroughbred or other stock, and, in the meantime we strongly urge our State authorities to quarantine against other States, and allow no cattle to be shipped into this State for breeding or dairy purposes unless accompanied by a clean bill of health, based on the tuberculin-test, or to have them tested by a veterinarian satisfactory to the board of control at point of entry at owner's expense."

The next subject brought before the Association was the Antivivisection Bill, which was introduced in the last Congress. After the subject had been fully discussed the following resolutions were adopted: "*Whereas*, there is before the Senate of the United States a bill which would check all vivisection and experiments directed toward the amelioration of disease among the lower animals and men; and, *whereas*, such experiments as are conducted by the Bureau of Animal Industry are of incalculable value to the live-stock industry of the country; and, *whereas*, this bill in our opinion is a misdirected philanthropy, which tends to protect the few at the expense of the many; be it *Resolved*, that the Virginia State Veterinary Medical Association request the members of the Senate and House of Representatives from this State to oppose to their utmost any measure tending to curtail the usefulness of the Department of Agriculture in the District of Columbia in its investigations of the diseases of live-stock."

The report of the officers showed the Association to be in a most flourishing condition, and it now includes in its membership all of the legitimate veterinarians in the State, with the exception of three, and we hope to have them as members at our next meeting.

After adjourning, the Association, as a body, visited that fine old Virginia resort, Ocean View, and enjoyed the luxuries thereof.

The Association adjourned to meet at Staunton, Va., January 5, 1897, thereby bringing to a close one of the most successful meetings ever held by the Association.

GEORGE C. FAVILLE, D.V.M.,
Secretary.

THOMAS M. SWEENY, V.S., Richmond,
Secretary-elect.

W. H. HARBAUGH, V.S.,
President.

